# Spatial heterogeneity of hepatic fibrosis in primary sclerosing cholangitis vs. viral hepatitis assessed by MR elastography

**DOI:** 10.1038/s41598-021-89372-4

**Published:** 2021-05-10

**Authors:** Rolf Reiter, Mehrgan Shahryari, Heiko Tzschätzsch, Dieter Klatt, Britta Siegmund, Bernd Hamm, Jürgen Braun, Ingolf Sack, Patrick Asbach

**Affiliations:** 1 Department of Radiology, Charité – Universitätsmedizin Berlin, Corporate Member of Freie Universität Berlin and Humboldt-Universität zu Berlin, Charitéplatz 1, 10117 Berlin, Germany; 2 Berlin Institute of Health (BIH), Anna-Louisa-Karsch-Str. 2, 10178 Berlin, Germany; 3 Richard and Loan Hill Department of Bioengineering, University of Illinois At Chicago, 830 South Wood Street, Chicago, IL 60612 USA; 4 Department of Gastroenterology, Infectiology, Rheumatology, Charité – Universitätsmedizin Berlin, Corporate Member of Freie Universität Berlin and Humboldt-Universität zu Berlin, Hindenburgdamm 30, 12203 Berlin, Germany; 5 Department of Medical Informatics, Charité – Universitätsmedizin Berlin, Corporate Member of Freie Universität Berlin and Humboldt-Universität zu Berlin, Hindenburgdamm 30, 12203 Berlin, Germany

**Keywords:** Biomarkers, Hepatitis, Liver cirrhosis, Liver fibrosis, Primary sclerosing cholangitis, Primary sclerosing cholangitis, Biomarkers, Viral hepatitis

## Abstract

Spatial heterogeneity of hepatic fibrosis in primary sclerosing cholangitis (PSC) in comparison to viral hepatitis was assessed as a potential new biomarker using MR elastography (MRE). In this proof-of-concept study, we hypothesized a rather increased heterogeneity in PSC and a rather homogeneous distribution in viral hepatitis. Forty-six consecutive subjects (PSC: *n* = 20, viral hepatitis: *n* = 26) were prospectively enrolled between July 2014 and April 2017. Subjects underwent multifrequency MRE (1.5 T) using drive frequencies of 35–60 Hz and generating shear-wave speed (SWS in m/s) maps as a surrogate of stiffness. The coefficient of variation (CV in %) was determined to quantify fibrosis heterogeneity. Mean SWS and CV were 1.70 m/s and 21% for PSC, and 1.84 m/s and 18% for viral hepatitis. Fibrosis heterogeneity was significantly increased for PSC (*P* = 0.04) while no difference was found for SWS of PSC and viral hepatitis (*P* = 0.17). Global hepatic stiffness was similar in PSC and viral hepatitis groups, but spatial heterogeneity may reveal spatial patterns of stiffness changes towards enhanced biophysics-based diagnosis by MRI.

## Introduction

Primary sclerosing cholangitis (PSC) is a chronic liver disease that leads to multifocal inflammation and stricturing of bile ducts. This progressive process causes fibrosis with a heterogeneous distribution pattern in comparison to other chronic liver diseases such as viral hepatitis^1–7^. Currently, there is no effective medical treatment besides liver transplantation. While magnetic resonance cholangiopancreatography allows noninvasive detection of PSC, determination of disease severity remains challenging^1,2,6–8^. Early efforts have been made for an MRI-based assessment of disease severity in PSC. For instance, Ruiz et al. developed progression risk scores based on the overall radiologic course as primary endpoint^9^. Parenchymal enhancement heterogeneity was one of the predictive radiologic features and an association with radiologic progression with an area under the curve (AUC) between 0.80 and 0.83 was found^9^. Moreover, Khoshpouri et al. showed liver and spleen volumetries correlate with PSC disease severity as determined by the Mayo risk score^10^ and predict transplant–free survival^11,12^. Nevertheless, even biopsy is inaccurate in PSC due to nonspecific histopathological features, considerable variation, and a heterogeneous distribution throughout the liver^1,13^. This lack of an accurate diagnostic reference standard to monitor disease activity limits patient management, stratification for clinical trials, and development of new therapies.

Magnetic resonance elastography (MRE) is a noninvasive imaging method for measuring viscoelastic tissue properties^14–17^. It is currently being discussed as a potential surrogate marker for the assessment of hepatic fibrosis in PSC. Recently published data suggest that MRE can accurately detect the presence of cirrhosis and predict long-term patient outcomes in PSC^18–20^. Tomoelastography is an advanced multifrequency MRE technique that provides full-field-of-view elastograms of the entire liver with improved detail resolution^21^. It relies on compound multifrequency processing, is noise robust, and—given its high spatial resolution—has potential to advance liver MRE towards regional assessment of mechanical tissue properties^21^. Parameters are shear-wave speed (SWS in m/s), which is determined as a surrogate marker of stiffness, and the phase angle of the complex modulus (*φ* in rad) which is related to the tissue’s viscous properties and is a surrogate of fluidity, where *φ* = 0 rad indicates pure solids and *φ* = π/2 rad pure fluids^22,23^.

### Aim

Based on reported visual impressions of experienced abdominal radiologists and pathologists, we hypothesize that fibrosis is heterogeneous in PSC and homogeneous in viral hepatitis^1,2,9^. To the best of our knowledge, this hypothesis has never been confirmed quantitatively by measuring viscoelastic tissue properties. Moreover, PSC and viral hepatitis can easily be distinguished using routine clinical testing and are thus well suited for comparison in this explorative study. Therefore, we conducted a study using MRE to evaluate the heterogeneity of hepatic fibrosis as a potential new quantitative biomarker.

## Methods

### Subjects

**Figure 1:**
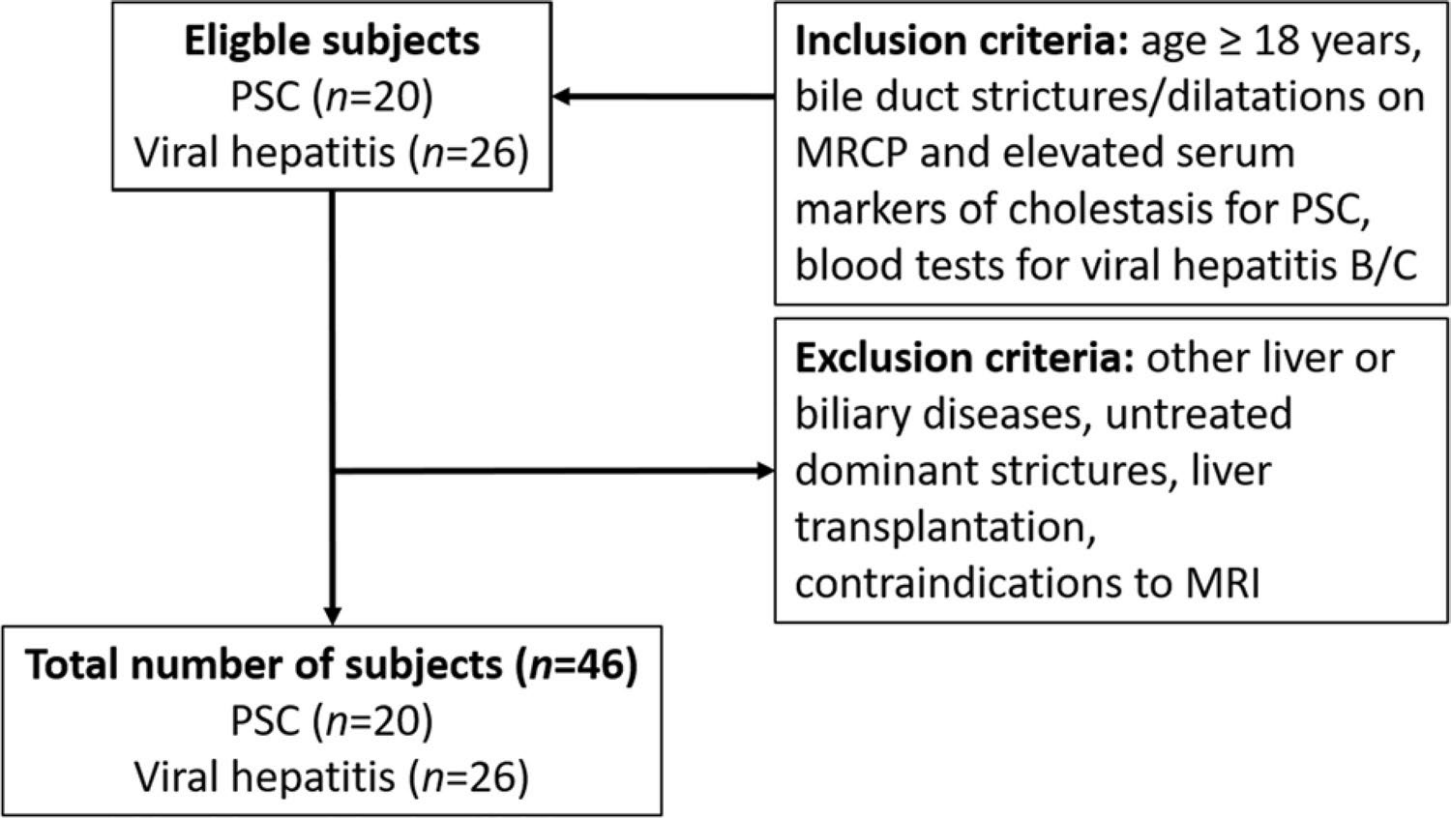
Flowchart of subjects. *PSC* primary sclerosing cholangitis, *MRCP* magnetic resonance cholangiopancreatography.

The study was approved by the institutional review board of the Charité—Universitätsmedizin Berlin and was conducted in accordance with relevant guidelines and regulations after obtaining oral and written patient informed consent. In this prospective single center study, we enrolled a total of 46 consecutive subjects between July 2014 and April 2017: 20 patients with PSC (mean age, 41.4 ± 14.1 years; 5 women) and 26 patients with viral hepatitis (mean age, 50.3 ± 14.5 years; 8 women). Inclusion criteria were multifocal bile duct strictures and segmental dilatations on magnetic resonance cholangiopancreatography in patients with elevated serum markers of cholestasis (alkaline phosphatase, γ-glutamyltransferase) for PSC, confirmation by routine laboratory blood testing for chronic viral hepatitis (e.g., surface antigen of the hepatitis B virus, hepatitis B core antigen, antibodies to the hepatitis C virus, and hepatitis C RNA), and age ≥ 18 years (Fig. 1)^13,24,25^. Exclusion criteria were other concurrent liver or biliary diseases, untreated dominant strictures (common bile duct ≤ 1.5 mm; left or right hepatic duct ≤ 1 mm) to avoid potential bias due to increased biliary pressure^19,26^, history of liver transplantation and general contraindications for magnetic resonance imaging. Additionally, as liver biopsy is not suited to stage PSC-related hepatic fibrosis, two biological scores were used to further characterize subjects: the aspartate aminotransferase-to-platelet ratio index (APRI) and the Mayo risk score (MRS). APRI is a surrogate marker to assess the severity of disease in relation to the stage of hepatic fibrosis and is based on routinely available laboratory test results^27^. Hepatic fibrosis is assessed as follows: APRI < 0.5, absence of significant fibrosis; APRI 0.5–1.0, cirrhosis unlikely; APRI 1.0–1.5, no reliable assessment; APRI 1.5–2.0, presence of significant fibrosis; and APRI > 2.0, cirrhosis. MRS is a surrogate marker of PSC long-term disease outcome and transplant-free survival^28^. The index incorporates age, variceal bleeding, serum albumin, aspartate aminotransferase, and bilirubin, and distinguishes three risk groups: MRS ≤ 0, low risk; 0 < MRS < 2, intermediate risk; and MRS > 2, high risk. All subjects fasted at least 4 h before MRE.

### Magnetic resonance elastography

Patients underwent multifrequency MRE with tomoelastography postprocessing at 1.5 T (Magnetom Aera, Siemens Healthineers, Germany) with an 18-channel phased-array coil in combination with the spine-array coil. The multifrequency MRE setup and components used in this study are described in detail in Hudert et al.^22^ and Shahryari et al.^23^. Briefly, subjects were placed in supine position and a custom-designed piezoelectric driver was positioned at the level of the xiphoid process. Mechanical vibrations at drive frequencies of 35, 40, 45, 50, 55 and 60 Hz were sequentially applied, and image acquisition was performed for a total of 4:30 min with patients breathing freely^29^. MRE imaging parameters were as follows: 9 axial slices, 8 offsets, full wave field, 300 × 234 mm^2^ field of view (fixed), 100 × 78 matrix size, 3 × 3 × 5 mm^3^ resolution, 50 Hz motion-encoding gradient frequency, 30 mT/m motion-encoding gradient amplitude, TR 1610 ms, TE 54 ms, GRAPPA factor 2, and 2 averages. Furthermore, the scan protocol included the following conventional sequences: axial T1-weighted dual gradient-echo sequence for liver fat quantification^30^, axial and coronal T2-weighted half-Fourier acquisition single-shot turbo spin-echo sequence, and, for PSC patients, magnetic resonance cholangiopancreatography using paracoronal T2-weighted single-shot turbo spin-echo sequences in thin-slice multisection (3 mm) and thick-slab (40–60 mm) acquisition.

### Data processing

Full-field-of-view elastograms—stiffness and fluidity maps—were computed based on 3D compound multifrequency processing^21^. The postprocessing pipeline is publicly available at https://bioqic-apps.charite.de. Each elastogram slice was generated by compounding 216 images of multifrequency MRE raw data using 12 spatiotemporal filter directions, 3 field components, and 6 drive frequencies. Using a systematic approach, volumes of interest were manually drawn by one radiologist who was blinded to clinical parameters (R.R., 9 years of experience in abdominal MRE) by contouring livers on SWS maps. Major blood vessels and regions of insufficient wave excitation were consistently excluded by using a lower SWS threshold of 1 m/s, as previously described^31^. The same volume of interest was used for both SWS and fluidity maps. Fibrosis was staged using cutoff values as previously published: F1 (any fibrosis) ≥ 1.52 m/s, F2 (moderate fibrosis) ≥ 1.55 m/s, F3 (severe fibrosis) ≥ 1.67 m/s, and F4 (cirrhosis) ≥ 1.72 m/s^31^. For quantifying fibrosis heterogeneity, we assessed intrahepatic standard deviations (SD) of SWS and *φ*. Results were normalized by dividing individual intrahepatic SDs with their corresponding means to account for differences in fibrosis severity between the two groups, yielding the coefficient of variation (CV in %).

### Statistical data analysis

Group values were calculated as mean and interindividual SD. A two-sided t-test and Pearson's correlation coefficient were used to assess MRE parameters and biological scores. AUC analysis with 95% confidence intervals was used to assess diagnostic performance of CV. The level of significance was *P* < 0.05. Statistical analysis was conducted using Matlab version 9.0 R2016a (The Mathworks, Inc., United States).

## Results

### Magnetic resonance elastography

**Figure 2:**
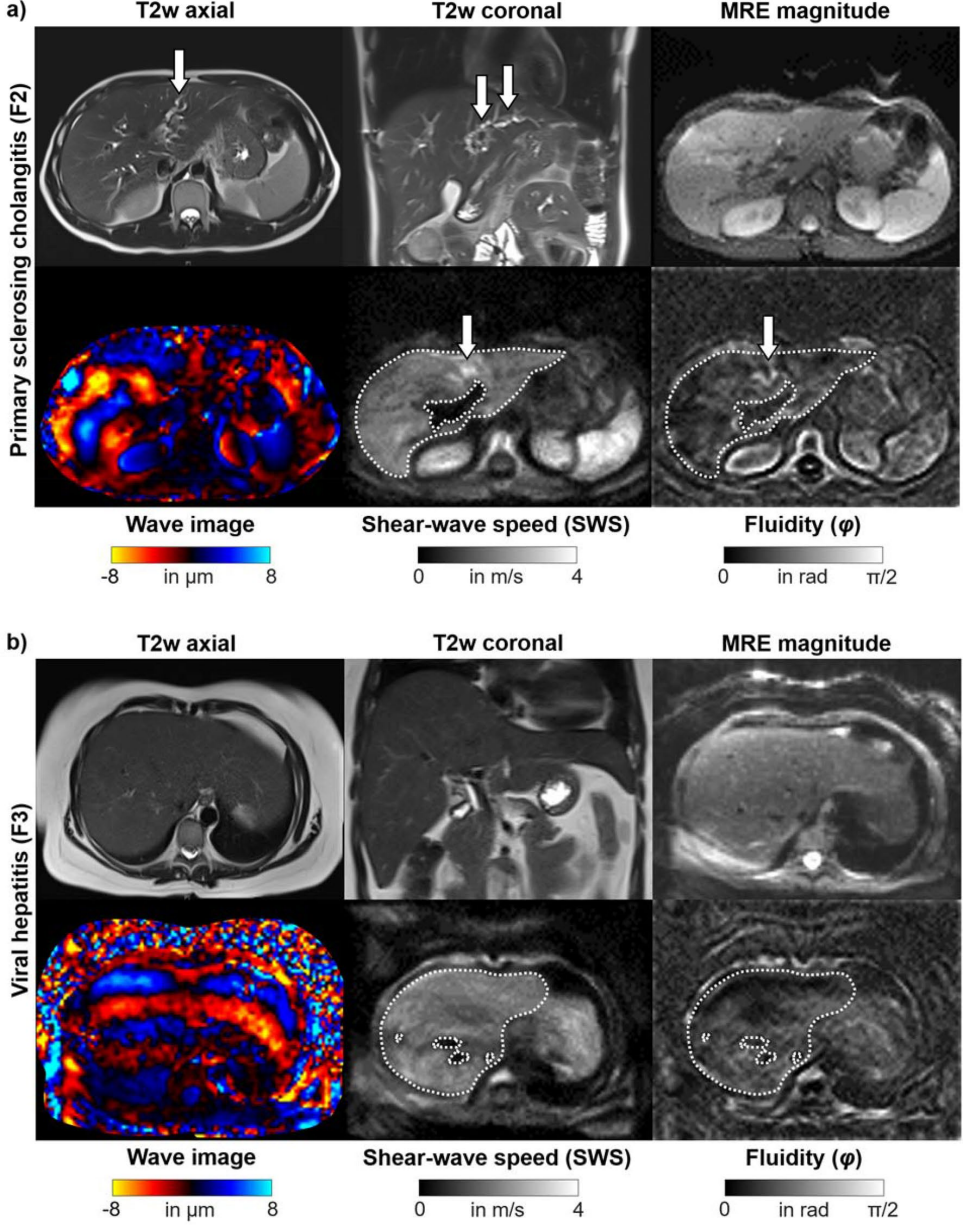
Representative cases. (**a**) 36-year-old man with primary sclerosing cholangitis (PSC) and moderate fibrosis (stage F2). There is irregular intrahepatic biliary dilatation in segments II and IV (arrows) on axial and coronal T2w images. MRE magnitude: morphological magnitude image derived from the magnetic resonance elastography (MRE) sequence. The wave image displays tissue displacement into and out of the axial plane. The full-field-of-view elastograms are quantitative grayscale maps of shear-wave speed (SWS) and fluidity (*φ*). In the region of interest (white dashed lines), the patient shows SWS and *φ* values with corresponding coefficient of variation (CV) of 1.56 m/s and 18%, and 0.62 rad and 32%, respectively. A rather heterogeneous PSC-related fibrosis distribution with a focal increase in stiffness in the area of irregular biliary dilatation in segment II and IV is visually apparent (arrows). (**b**) 32-year-old woman with viral hepatitis B with SWS and *φ* values with corresponding CV of 1.69 m/s and 17%, and 0.45 rad and 41%, respectively. Despite severe fibrosis (stage F3), both the stiffness and the fluidity map show a rather homogenous viral hepatitis-related fibrosis distribution.

**Figure 3:**
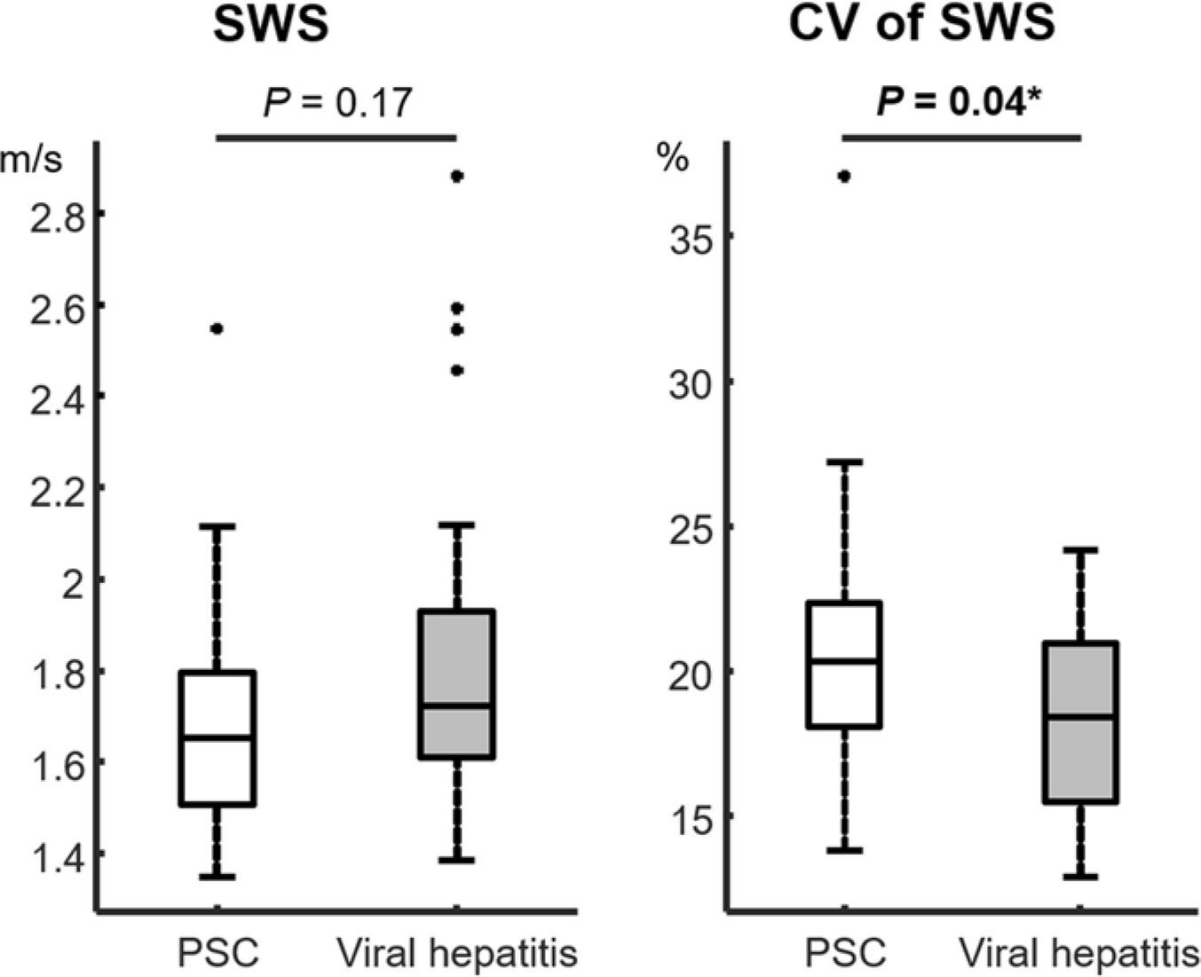
Boxplots display median, upper and lower quartiles, and whiskers of shear-wave speed (SWS) and coefficient of variation (CV). Statistically significant differences are indicated by an asterisk (*) for *P* < 0.05. *PSC* primary sclerosing cholangitis**.**

Figure 2 shows representative cases with a rather heterogeneous fibrosis distribution in PSC and a rather homogeneous distribution in viral hepatitis. Extended baseline characteristics including biological scores and laboratory results are listed in Table 1, and MRE parameters are listed in Table 2. Fibrosis stage distributions based on MRE were as follows: for PSC: F0, *n* = 6; F1, *n* = 2; F2, *n* = 2; F3, *n* = 1; and F4, *n* = 9; and for viral hepatitis (hepatitis B, *n* = 15; hepatitis C, *n* = 11): F0, *n* = 4; F1, *n* = 2; F2, *n* = 4; F3, *n* = 3; and F4, *n* = 13. According to this distribution, the resulting mean fibrosis stage was 2.25 in PSC and 2.73 in viral hepatitis. Mean volume of interest was 561.7 ± 179.6 cm^3^. A significant increase of mean values of CV of SWS was found for PSC vs. viral hepatitis (21% vs. 18%; *P* = 0.04) but not for SWS (1.70 m/s vs. 1.84 m/s; *P* = 0.17) (Fig. 3). In a subgroup analysis of F0–F2 patients, a trend for increased mean values of CV of SWS was found for PSC vs. viral hepatitis without reaching statistical significance (19% vs. 17%; *P* = 0.086). All patients with visual signs of cirrhosis showed SWS ≥ 1.72 m/s (F4 cutoff value), whereas only some patients (PSC, *n* = 4; viral hepatitis, *n* = 6) with MRE-based F4 stage showed visual signs of cirrhosis. In the category of F4 patients, mean SWS was significantly increased for PSC patients with vs. without segmental atrophy/hypertrophy (2.13 m/s vs. 1.77 m/s, *P* = 0.04), whereas no significant difference was found for viral hepatitis (2.27 m/s vs. 1.98 m/s, *P* = 0.18). For both PSC and viral hepatitis, a strong correlation between SWS and SD of SWS (*R* = 0.87, 0.93; with *P* ≤ 0.008, respectively) and between SWS and CV of SWS (*R* = 0.63, 0.62; with *P* ≤ 0.003, respectively) was found. For *φ* data, there was no significant difference between PSC and viral hepatitis (*φ* and CV of *φ* with *P* = 0.64, 0.75; respectively). A preliminary assessment of diagnostic performance showed AUC values (95% confidence interval) of 0.65 (0.51–0.76) for CV of SWS, and 0.50 (0.35–0.63) for CV of *φ*.

**Table 1:** Baseline characteristics of subjects.

	All subjects	PSC	Viral hepatitis
	*n* = 46	*n* = 20	*n* = 26
Age (years)	46 (15)	41 (14)	50 (14)
Female (%)	28	25	31
Body mass index (kg/m^2^)	24 (4)	23 (4)	25 (4)
Liver Fat Content (%)	4 (5)	3 (3)	4 (6)
APRI Score	1.84 (4.13)	0.99 (1.73)	2.5 (5.19)
Mayo Risk Score	–	− 1.97 (7.95)	–
ALP (U/I)	158.12 (122.44)	219.50 (126.29)	70.43 (23.50)
γGT (U/I)	212.66 (276.56)	287.99 (329.35)	118.50 (143.32)
Bilirubin (mg/dl)	1.35 (2.32)	1.64 (3.11)	1.03 (0.63)
ALT (U/I)	82.00 (65.22)	86.35 (59.84)	76.56 (71.01)
AST (U/I)	74.50 (84.02)	64.05 (56.46)	82.54 (99.44)
Transaminases > 5–10 × ULN (number of pt.)	6	3	3
Liver surface nodularity (number of pt.)	10	4	6
Segmental atrophy/hypertrophy (number of pt.)	10	4	6
Ascites (number of pt.)	9	5	4
Collateral vessels/varices (number of pt.)	3	2	1
Splenomegaly (> 12 cm, number of pt.)	13	5	8
Spleen size (cm)	11.78 (2.67)	11.79 (2.82)	11.78 (2.54)

If not otherwise specified, data are means and numbers in parentheses are standard deviations.

*APRI* aspartate aminotransferase-to-platelet ratio index, *ALP* alkaline phosphatase, *γGT* gamma-glutamyltransferase, *ALT* alanine transaminase, *AST* aspartate transaminase, *ULN* upper limit of normal.

**Table 2:** MRE results.

	All subjects	PSC	Viral hepatitis
	*n* = 46	*n* = 20	*n* = 26
SWS (m/s)	1.78 (0.35)	1.70 (0.28)	1.84 (0.38)
SD of SWS (m/s)	0.35 (0.13)	0.36 (0.14)	0.35 (0.13)
CV of SWS (%)	19 (4)	21 (5)	18 (3)
*φ* (rad)	0.49 (0.12)	0.48 (0.10)	0.50 (0.13)
SD of *φ* (rad)	0.20 (0.03)	0.20 (0.03)	0.20 (0.03)
CV of *φ* (%)	41 (7)	42 (5)	41 (7)
Fibrosis stage	2.52 (1.64)	2.25 (1.76)	2.73 (1.51)

Data are means and numbers in parentheses are standard deviations.

*SWS* shear-wave speed, *SD* standard deviation, *CV* coefficient of variation, *φ* phase angle of the complex shear modulus.

### Biological scores

For PSC, there was a significant correlation between APRI and SWS (*R* = 0.60, *P* = 0.005), which even further increased between APRI and CV of SWS (*R* = 0.85, *P* = 0.02). For MRS, no correlation was found in the PSC group for SWS, CV of SWS and *φ*, but a moderate negative correlation with CV of *φ* (*R* = − 0.46, *P* = 0.05). Consistent with results for mean SWS values, the not statistically significant trend towards higher fibrosis severity in the viral hepatitis group was also reflected by APRI with mean values of 2.5 ± 5.19 for viral hepatitis and 0.99 ± 1.73 for PSC (*P* = 0.23). For PSC, the MRS group mean was − 1.97 ± 7.95, indicating low risk.

## Discussion

We conducted a study using MRE to evaluate the spatial heterogeneity of hepatic fibrosis as a potential new biomarker. Our results quantitatively confirm known visual impressions of experienced abdominal radiologists and pathologists^1,2,9^ by demonstrating increased stiffness-based heterogeneity in PSC as compared to viral hepatitis where a rather homogenous fibrosis distribution was found. The CV of MRE-based stiffness has the potential to quantify heterogeneity in PSC-related hepatic fibrosis as a new biomarker besides global stiffness, whereas the CV of fluidity was not found to be sensitive.

Despite a tendency towards higher mean SWS in the viral hepatitis group, a significantly increased fibrosis heterogeneity was found in the PSC group. However, only a moderate diagnostic performance was found in this explorative study. For PSC and viral hepatitis, an increase in both SD and CV of SWS with the severity of fibrosis was shown. This relationship has been reported previously for SD^31–33^. Presumably, this correlation is based on unevenly distributed structural changes of extracellular matrix components occurring during fibrogenesis, such as the development of regenerating nodules, synthesis and connection of free collagen branches, and architectural distortions in the focal area of cell damage^34–36^. Our results also reproduce the correlation between mean stiffness measured by MRE or ultrasound elastography and APRI reported by earlier investigations^32,35^. It is a stimulating result of our study that, compared to mean SWS values, an even stronger correlation has emerged between APRI and the CV of SWS in the PSC group but not in the viral hepatitis group. This finding might indicate an impact of heterogeneity on the stiffness-based staging of PSC-related fibrosis. However, larger studies are necessary to confirm this preliminary finding and to determine the potential diagnostic benefit for the assessment of disease activity or prognosis of PSC. The lack of correlation with MRS, except for CV of *φ*, might be attributable to the fact that the MRS is a statistical model for predicting long-term disease outcome rather than characterizing the current status of hepatobiliary disease activity. Another contributing factor might be the low risk profile of our study population based on MRS.

Habibabadi et al. reported initial experience in detecting heterogeneity of hepatic fibrosis^37^. They investigated 128 patients with a wide range of underlying chronic liver diseases and suspected fibrosis using MRE. Heterogeneity of fibrosis was defined as present when the first and the second most predominant fibrosis stages were more than one category apart. Comparing region-of-interest-based and volumetric measurements, they found that global liver stiffness may not represent the entire spectrum of hepatic fibrosis^37^. Jhaveri et al. validated MRE in a group of 67 PSC patients using transient elastography as reference standard. For the differentiation of early and moderate fibrosis from cirrhosis, they found a good diagnostic performance with a sensitivity, specificity and accuracy of 87.5%, 96.1% and 94.0%, respectively^18^. Bookwalter et al. showed a low significant correlation between the stiffness of liver segments and corresponding segmental bile duct strictures in a group of 55 PSC patients (*R* = 0.18, *P* < 0.001)^19^. At the same time, they did not find a correlation between stiffness and conventional magnetic resonance cholangiopancreatography findings such as segmental bile duct dilatation, thickening or enhancement. In alignment with our study, no correlation between stiffness and MRS was found. However, published results on the relationship of stiffness and MRS are mixed^18–20,38,39^. For instance, Idilman et al. observed a positive correlation between hepatic stiffness and MRS (*R* = 0.646, *P* < 0.0001) in a large cohort of 266 patients^39^. Moreover, Tafur et al. found that stiffness had a superior discriminatory ability than morphological intrahepatic stricture severity to distinguish MRS-based risk groups (AUC of 0.779 and 0.718; respectively)^38^. In contrast to our study, they found a moderately positive correlation between stiffness and MRS (*R* = 0.6; *P* ≤ 0.001), which may be related to an increased median MRS group value of − 0.07 in their study versus − 0.50 in our study. However, a moderately negative correlation between CV of *φ* and MRS was found in our study (*R* =  − 0.45; *P* = 0.05). Corpechot et al. performed a study using transient elastography in 73 PSC patients with biopsy-proven fibrosis^40^. They found a good diagnostic performance of transient elastography with accuracy values for identification of severe fibrosis and cirrhosis of 83% and 88%, respectively^40^. Moreover, they reported the diagnostic performance of transient elastography to be superior to APRI and MRS in patients with significant or severe fibrosis.

Of potential interest for the further development of heterogeneity as a promising new biomarker in MRE might be the so-called 1-Norm technique, which is based on a mathematical characterization of shear wave front geometry^41^. It provides a quantitative measure of the magnitude of wave scattering, which can potentially be used as a (in-)homogeneity index of biological tissue. This method was not available for in vivo application at the time of our study but will be implemented for future work. Moreover, also the assessment of heterogeneity of mapping and diffusion parameters should be investigated in the future.

Although encouraging, our study has limitations. First, there is no accurate reference standard available for the evaluation of disease activity in PSC, which in turn motivated this study. Fibrosis staging was based on MRE thresholds that have been established in a cohort of patients with mixed underlying chronic liver diseases^31^. Second, we investigated a small number of patients, especially for fibrosis stages F1–F3, as PSC is a rare disease. However, the preliminary results of this explorative study motivate further investigation of heterogeneity in hepatic fibrosis by elastography, and larger studies to confirm our findings should be performed. Third, some subjects were included in a previous study to investigate the diagnostic performance of MRE for staging hepatic fibrosis (nine patients with PSC and eleven patients with viral hepatitis)^31^. Finally, measuring CV of stiffness might not necessarily mean measuring heterogeneity of fibrosis given the fact that stiffness is an indirect measure of fibrosis which can be confounded by many factors including portal hypertension^42^ and inflammation^43^. Nonetheless, this study is a first step towards quantitatively relating fibrosis heterogeneity to PSC.

In conclusion, our results show an increased stiffness-based heterogeneity in PSC as compared to viral hepatitis. Global hepatic stiffness was similar in PSC and viral hepatitis groups, but spatial heterogeneity may reveal spatial patterns of stiffness changes towards enhanced biophysics-based diagnosis by MRI.

## Data Availability

The datasets used and/or analysed during the current study are available from the corresponding author on reasonable request pending approval by the local data security authorities.

## References

[1] Burak K (2003). Is there a role for liver biopsy in primary sclerosing cholangitis?. Am. J. Gastroenterol..

[2] Zenouzi R, Welle CL, Venkatesh SK, Schramm C, Eaton JE (2019). Magnetic resonance imaging in primary sclerosing cholangitis—current state and future directions. Semin. Liver Dis..

[3] Yang M (2020). Incidence and risk factors of hepatocellular carcinoma in patients with hepatitis C in China and the United States. Sci. Rep..

[4] Gorin J-B (2020). Plasma FABP4 is associated with liver disease recovery during treatment-induced clearance of chronic HCV infection. Sci. Rep..

[5] Ferrasi AC (2020). New LncRNAs in chronic hepatitis C progression: from fibrosis to hepatocellular carcinoma. Sci. Rep..

[6] Khoshpouri P (2019). Imaging features of primary sclerosing cholangitis: from diagnosis to liver transplant follow-up. Radiographics.

[7] Ito K, Mitchell DG, Outwater EK, Blasbalg R (1999). Primary sclerosing cholangitis: MR imaging features. Am. J. Roentgenol..

[8] Bader TR, Beavers KL, Semelka RC (2003). MR imaging features of primary sclerosing cholangitis: patterns of cirrhosis in relationship to clinical severity of disease. Radiology.

[9] Ruiz A (2014). Radiologic course of primary sclerosing cholangitis: assessment by three-dimensional magnetic resonance cholangiography and predictive features of progression. Hepatology.

[10] Khoshpouri P (2018). Correlation between quantitative liver and spleen volumes and disease severity in primary sclerosing cholangitis as determined by Mayo risk score. Eur. J. Radiol..

[11] Khoshpouri P (2019). Quantitative spleen and liver volume changes predict survival of patients with primary sclerosing cholangitis. Clin. Radiol..

[12] Khoshpouri P (2020). Cross-sectional imaging in patients with primary sclerosing cholangitis: single time-point liver or spleen volume is associated with survival. Eur. J. Radiol..

[13] European Association for the Study of the Liver (2009). EASL clinical practice guidelines: management of cholestatic liver diseases. J. Hepatol..

[14] Muthupillai R (1995). Magnetic resonance elastography by direct visualization of propagating acoustic strain waves. Science.

[15] Guo J (2014). Patient-activated three-dimensional multifrequency magnetic resonance elastography for high-resolution mechanical imaging of the liver and spleen. RoFo.

[16] Kamphues C (2012). Viscoelasticity-based magnetic resonance elastography for the assessment of liver fibrosis in hepatitis C patients after liver transplantation. RoFo.

[17] Klatt D (2008). Assessment of the solid–liquid behavior of the liver for the diagnosis of diffuse disease using magnetic resonance elastography. RoFo.

[18] Jhaveri KS (2019). The development and validation of magnetic resonance elastography for fibrosis staging in primary sclerosing cholangitis. Eur. Radiol..

[19] Bookwalter CA, Venkatesh SK, Eaton JE, Smyrk TD, Ehman RL (2018). MR elastography in primary sclerosing cholangitis: correlating liver stiffness with bile duct strictures and parenchymal changes. Abdom. Radiol..

[20] Eaton JE (2016). Performance of magnetic resonance elastography in primary sclerosing cholangitis. J. Gastroenterol. Hepatol..

[21] Tzschätzsch H (2016). Tomoelastography by multifrequency wave number recovery from time-harmonic propagating shear waves. Med. Image Anal..

[22] Hudert CA (2019). Tomoelastography for the evaluation of pediatric nonalcoholic fatty liver disease. Investig. Radiol..

[23] Shahryari M (2019). Tomoelastography distinguishes noninvasively between benign and malignant liver lesions. Cancer Res..

[24] Chapman R (2010). Diagnosis and management of primary sclerosing cholangitis. Hepatology.

[25] Eaton JE, Talwalkar JA, Lazaridis KN, Gores GJ, Lindor KD (2013). Pathogenesis of primary sclerosing cholangitis and advances in diagnosis and management. Gastroenterology.

[26] Björnsson E, Lindqvist-Ottosson J, Asztely M, Olsson R (2004). Dominant strictures in patients with primary sclerosing cholangitis. Am. J. Gastroenterol..

[27] Wai CT (2003). A simple noninvasive index can predict both significant fibrosis and cirrhosis in patients with chronic hepatitis C. Hepatology.

[28] Kim WR (2000). A revised natural history model for primary sclerosing cholangitis. Mayo Clin. Proc..

[29] Shahryari M (2021). Reduction of breathing artifacts in multifrequency magnetic resonance elastography of the abdomen. Magn. Reson. Med..

[30] Fischer MA (2012). Liver fat quantification by dual-echo MR imaging outperforms traditional histopathological analysis. Acad. Radiol..

[31] Reiter R (2020). Diagnostic performance of tomoelastography of the liver and spleen for staging hepatic fibrosis. Eur. Radiol..

[32] Huwart L (2007). Liver fibrosis: noninvasive assessment with MR elastography versus aspartate aminotransferase–to-platelet ratio index. Radiology.

[33] Venkatesh SK, Wang G, Lim SG, Wee A (2014). Magnetic resonance elastography for the detection and staging of liver fibrosis in chronic hepatitis B. Eur. Radiol..

[34] Reiter R (2014). Wideband MRE and static mechanical indentation of human liver specimen: sensitivity of viscoelastic constants to the alteration of tissue structure in hepatic fibrosis. J. Biomech..

[35] Rockey DC, Bissell DM (2006). Noninvasive measures of liver fibrosis. Hepatology.

[36] Ratziu V (2005). Sampling variability of liver biopsy in nonalcoholic fatty liver disease. Gastroenterology.

[37] Rezvani Habibabadi R (2020). Comparison between ROI-based and volumetric measurements in quantifying heterogeneity of liver stiffness using MR elastography. Eur. Radiol..

[38] Tafur M (2020). Risk stratification in primary sclerosing cholangitis: comparison of biliary stricture severity on MRCP versus liver stiffness by MR elastography and vibration-controlled transient elastography. Eur. Radiol..

[39] Idilman IS, Low HM, Bakhshi Z, Eaton J, Venkatesh SK (2020). Comparison of liver stiffness measurement with MRE and liver and spleen volumetry for prediction of disease severity and hepatic decompensation in patients with primary sclerosing cholangitis. Abdom. Radiol..

[40] Corpechot C (2014). Baseline values and changes in liver stiffness measured by transient elastography are associated with severity of fibrosis and outcomes of patients with primary sclerosing cholangitis. Gastroenterology.

[41] Palnitkar H (2019). An investigation into the relationship between inhomogeneity and wave shapes in phantoms and ex vivo skeletal muscle using magnetic resonance elastography and finite element analysis. J. Mech. Behav. Biomed. Mater..

[42] Zhao Z-L (2020). Author correction: Imaging and pathological features of idiopathic portal hypertension and differential diagnosis from liver cirrhosis. Sci. Rep..

[43] Kim JW (2020). Multiparametric MR index for the diagnosis of non-alcoholic steatohepatitis in patients with non-alcoholic fatty liver disease. Sci. Rep..

